# Tenascin-c knockdown suppresses vasculogenic mimicry of gastric cancer by inhibiting ERK- triggered EMT

**DOI:** 10.1038/s41419-021-04153-1

**Published:** 2021-09-29

**Authors:** Xing Kang, En Xu, Xingzhou Wang, Lulu Qian, Zhi Yang, Heng Yu, Chao Wang, Chuanfu Ren, Yizhou Wang, Xiaofeng Lu, Xuefeng Xia, Wenxian Guan, Tong Qiao

**Affiliations:** 1 Department of General Surgery, Nanjing Drum Tower Hospital Clinical College of Nanjing Medical University, No. 321 Zhongshan Road, 210008 Nanjing, China; 2 Department of General Surgery, Affiliated Drum Tower Hospital, Medical School of Nanjing University, No. 321 Zhongshan Road, 210008 Nanjing, China; 3 Department of Vascular Surgery, The Affiliated Drum Tower Hospital of Nanjing University Medical School, No. 321 Zhongshan Road, 210008 Nanjing, China

**Keywords:** Gastric cancer, Tumour angiogenesis

## Abstract

Gastric cancer is one of the most common malignancies worldwide and vasculogenic mimicry (VM) is considered to be the leading cause for the failure of anti-angiogenesis therapy in advanced gastric cancer patients. In the present study, we investigate the role of tenascin-c (TNC) in the formation of VM in gastric cancer and found that TNC was upregulated in gastric cancer tissue than in the corresponding adjacent tissues and correlated with VM and poor prognosis of gastric cancer. Furthermore, knockdown of TNC significantly inhibited VM formation and proliferation of gastric cancer cells in vitro and in vivo, with a reduction in cell migration and invasion. Mechanistically, TNC knockdown suppressed the phosphorylation of ERK and subsequently inhibited the process of EMT, both of which play an important role in VM formation. Our results indicated that TNC plays an important role in VM formation in gastric cancer. Combining inhibition of TNC and ERK may be a potential therapeutic approach to inhibit gastric cancer growth and metastasis and decrease antiangiogenic therapeutic resistance.

## Introduction

Gastric cancer is a malignant tumor originating from the mucosal epithelium of stomach. In China, the incidence of gastric cancer ranks second and the mortality rate ranks third [1]. Approximately 1.2 million new cases of gastric cancer occur each year worldwide, and China accounts for about 40% [2, 3]. The proportion of early gastric cancer is low (about 40%), and most of them are already advanced (about 60%) when diagnosed, due to lack of early clinical symptoms [4]. The treatment options for advanced gastric cancer are few and the efficacy is limited and the 5-year survival rate is <20% [5]. Even though the diagnosis and treatment of gastric cancer have improved these years, the prognosis of patients is still poor. Gastric cancer recurrence and metastasis remain the most common cause of poor long-term survival [6]. Tumor growth and metastasis require an adequate blood supply. However, antivascular therapy did not improve the survival in patients with advanced gastric cancer. In addition, antiangiogenic therapy has been hypothesized to induce the formation of vasculogenic mimicry (VM) [7], without the participation of endothelial cells in the formation of the blood supply pattern. Thus, VM may play an important role in the development of GC and may be a major cause of failure in antiangiogenic therapy.

VM was first discovered in melanoma in 1999 [8]. It is a vascular-like structure that can mimic the embryonic vascular network pattern to nourish the tumor tissue [9]. The structure formed by VM is composed of a basement membrane and lined by cancer cells without endothelial cells on the inner wall, allowing blood plasma and red blood cells to flow [10]. VM structure serves as an adjunct to the vasculature system, helping tumor to grow. Thus, due to direct exposure to the blood, cancer cells can easily leak out and migrate through the bloodstream to metastasize to other organs [11]. Recent studies have shown that VM is observed and associated with poor prognosis in many malignant tumors, including breast cancer [12], hepatocellular cancer [13], osteosarcoma [14], colorectal cancer [15] and gastric cancer [16, 17]. However, few studies have considered VM as a potential therapeutic target in gastric cancer, and the mechanisms underlying VM remain unknown.

Tenascin-C (TNC) is a hexameric, multimodular extracellular matrix protein and is the founding member of the tenascin gene family [18]. The synthesis of TNC is tightly regulated and the distribution of TNC is highly restricted in adult tissues. However, it is specifically upregulated during wound healing, chronic inflammation or cancer [19]. Recent studies have shown that the expression of TNC was significantly associated with pT stage, lymph node metastasis and distant metastasis in gastric cancer [20]. TNC was identified in inducing epithelial-to-mesenchymal (EMT) transition [21] and EMT has been found to play a key role in VM formation [9, 22, 23]. However, the role of TNC in VM formation and EMT in gastric cancer was not reported.

In this study, we therefore investigated the role of TNC in VM formation in gastric cancer cell lines. We used siRNA to knock down the expression of TNC and evaluated the changes in VM formation and EMT markers. Our findings suggest that TNC may represent a novel target in the treatment of gastric cancer.

## Materials and methods

### Clinical samples

Fifty pairs of gastric cancer tissues and corresponding adjacent tissues and 66 gastric cancer samples from patients who underwent surgery more than 5 years were obtained from the General Surgery Department of the Nanjing Drum Tower Hospital. The research protocol complies with the ethical guidelines of the 1975 Declaration of Helsinki. All experiments with human samples have been reviewed and approved by the Ethics Committee and Animal Welfare Committee of Nanjing Drum Tower Hospital. Written informed consent was obtained from each participant before learning.

### Immunohistochemical (IHC) staining

The tissue is fixed in formalin and embedded in paraffin, and then cut into thin slices. The sections were deparaffinized with xylene and hydrated with ethanol, and then the antigen was recovered by pressure cooking. At room temperature, the sections were sliced with TNC primary antibody (ab108930, Abcam, Cambridge, MA), MMP9 (Abcam, ab76003, 1:1000 dilution), MMP2 (Abcam, ab92536, 1:1000 dilution) and CD31 (ab134168, Abcam), incubated for 60 min and then incubated with IgG H&L (HRP; 1:200 dilution, Abcam, Cambridge, UK). Then, the sections were stained with chromogen and counterstained with hematoxylin. The score is based on the intensity of staining (0 means no staining, 1 means weak staining, 2 means moderate staining, and 3 means strong staining). The product of the two levels was calculated as the final expression score.

### Cell culture

Three human gastric cancer cell lines, AGS, BGC-823 and Hs746T, were purchased from the American Type Culture Collection (Manassas, VA, USA). The AGS, BGC-823 and Hs746T cells were supplemented with 10% fetal bovine serum (FBS; Gibco, Waltham, MA, USA) in RPMI 1640 medium (Gibco, Waltham, MA, USA) and 1% penicillin/streptomycin (Gibco, Waltham, MA, USA). The medium was refreshed and the cells were passed every 3 days.

### siRNA transfection assay

According to the manufacturer’s instructions, GC cells were transfected with siRNA (Guangzhou Ribobio Co., Ltd.) specifically targeting TNC using interferon reagent (Polyplus, New York, USA). Firstly, 5 pmol of siRNA was diluted in 200 μL of medium without serum, vortexed for 20 s, and centrifuged briefly. Then, 8 μL of interferin reagent was added, vortexed for 20 s, centrifuged briefly, and then incubated for 10 min at room temperature. During the incubation time, 2 mL of serum-containing medium was prepared. Finally, the transfection mix was added to the cells in serum-containing medium. The siRNA target sequence is TAACCATTTCCGACATTAA. The knockdown efficiency of TNC siRNA was checked by western blot.

### Lentiviral transduction

Control lentivirus vectors and lentiviral vector encoding a gene-specific shRNA against TNC’s scrambled shRNA were purchased from Shanghai Gene-Pharma Co., Ltd (Shanghai, China). The human GC cell line was transduced with lentiviral particles along with polybrene, and screened with puromycin (1 mg/mL) (Thermo Fisher Scientific, Waltham, MA, USA) for 2 weeks. The knockdown efficiency was detected by western blot.

### Western blot analysis

The cell lysates were collected, and 30 mg protein of each sample was separated using 10% SDS-PAGE. The protein was then electrotransferred onto Polyvinylidene Fluoride (PVDF) membrane (Millipore, Boston, MA, USA). The PVDF membrane was sealed in 10% skimmed milk. Then the membrane was incubated with primary antibodies, including anti-TNC (Abcam, ab108930, 1:1000 dilution), anti-GAPDH (Abcam, ab181602, 1:10,000 dilution), and anti-ERK (Abcam, ab184699, 1:1000 dilution)), anti-p-ERK (Abcam, ab201015, 1:1000 dilution), anti-MMP9 (Abcam, ab76003, 1:1000 dilution), anti-MMP2 (Abcam, ab92536, 1:1000 dilution), anti-E-cadherin (Abcam, ab1416, 1:1000 dilution), anti-N-cadherin (Abcam, ab18203, 1:1000 dilution), anti-vimentin (Abcam, ab92547, 1:1000 dilution), anti-snail (Abcam, ab216347, 1:1000 dilution), anti-clumping (Abcam, ab27568, 1:1000 dilution) and anti-TWIST1 (Abcam, ab50887, 1:1000 dilution) overnight. Then the membrane and horseradish peroxidase-conjugated secondary antibody (Abcam AB6721, 1:10,000 dilution) were incubated for 2 h at room temperature. Chemiluminescence detection reagent (Millipore, Boston, MA, USA) was used to visualize protein bands.

### Reverse transcription quantitative real-time polymerase chain reaction (RT-qPCR)

Total RNA was extracted from GC cells, and then reverse transcribed into cDNA using an RT-PCR kit (Vazyme, China). Gene-specific primers were designed using Primer Express version 2.0 software (Applied Biosystems Inc., Milan, Italy), and are listed in Table 1. RT-PCR analysis was performed using SYBR Green Premix Ex Taq. Human GAPDH was used as an internal reference gene. The relative mRNA expression was calculated according to the 2^−ΔΔCt^ method.

**Table 1:** Primer used in quantitative real-time PCR assays.

Primer	Sequences (5′ → 3′)
GAPDH-F	GGAGTCCACTGGCGTCTTCA
GAPDH-R	GTCATGAGTCCTTCCACGATACC
TNC-F	TCCCAGTGTTCGGTGGATCT
TNC-R	TTGATGCGATGTGTGAAGACA
E-cadherin-F	CGAGAGCTACACGTTCACGG
E-cadherin-R	GGGTGTCGAGGGAAAAATAGG
N-cadherin-F	AGCCAACCTTAACTGAGGAGT
N-cadherin-R	GGCAAGTTGATTGGAGGGATG
Vimentin-F	GACGCCATCAACACCGAGTT
Vimentin-R	CTTTGTCGTTGGTTAGCTGGT
Slug-F	TGTGACAAGGAATATGTGAGCC
Slug-R	TGAGCCCTCAGATTTGACCTG
Snail-F	TCGGAAGCCTAACTACAGCGA
Snail-R	AGATGAGCATTGGCAGCGAG
Twist1-F	GTCCGCAGTCTTACGAGGAG
Twist1-R	GCTTGAGGGTCTGAATCTTGCT
MMP9-F	GGGACGCAGACATCGTCATC
MMP9-R	TCGTCATCGTCGAAATGGGC
MMP2-F	GATACCCCTTTGACGGTAAGGA
MMP2-R	CCTTCTCCCAAGGTCCATAGC

### Bioinformatics analysis

We used the ACRG database to analyze the relationship between TNC expression and the prognosis of gastric cancer. The gene expression data were derived from The Cancer Genome Atlas (TCGA; https://portal.gdc.cancer.gov/projects/TCGA-LIHC) and analyzed using Gene Set Enrichment Analysis (GSEA).

### Cell viability assays

The AGS, BGC-823 and Hs746T cell lines were seeded into 96-well microplates at a density of 5000 cells per well. The cell proliferation was determined using the Cell Counting Kit 8 (CCK-8) assay (Dojindo, Kumamoto, Japan) according to the manufacturer’s instructions. Briefly, 10 μL of LCCK-8 working solution was added per 100 μL of medium to the microtiter plate and the cells were incubated for 1.5 h. The OD450 value was determined by using the MRX II microplate reader (Dynex, Chantilly, VA, USA).

### EdU staining

The GC cells were incubated with 10 μM Edu (C0071L, Beyotime, Shanghai, PR China) for 2 h, and fixed with 4% paraformaldehyde for 15 min at room temperature. Then, the GC cells were washed with 3% Bovine serum albumin (BSA) in Phosphate Buffered Saline (PBS) 3 × 5 min, and containing 0.3% Triton PBS X-100 penetration for 10–15 min. The cells were washed thoroughly with PBS containing 3% BSA, and then incubated with Click Additive Solution for 30 min in the dark. Next, the cells were washed with PBS containing 3% BSA for 3 × 5 min, and then incubated with Hoechst 33342 for 10 min in the dark. Finally, the cells were washed and observed under an Olympus microscope.

### Cell cycle analysis

GC cells were treated with propidium iodide (PI; Dawen) and were analyzed by flow cytometry. Modfit Software (Verity Software House) was used for quantitative analysis of the cell cycle.

### Cell apoptosis analysis

The death of apoptotic cells was detected by flow cytometry using Annexin V-FITC Apoptosis Detection Kit II (KeyGEN BioTECH, Nanjing, China). The cells were harvested and washed twice with PBS. Then, cells were resuspended with 5 μL Annexin V-FITC and 500 μL1 × 5 μL PI binding buffer, and incubated for 15 min in the dark at room temperature. Then, the percentage of apoptotic cells was analyzed by flow cytometry.

### Wound-healing assay

The cells were seeded in a six-well plate at a density of 5 × 10^5^/well and cultured to 90% confluence. A plastic spatula was used to make a wound track on each plate, wash the plate with PBS to remove loose cell debris and replenish the plate with fresh low-serum culture medium (1% FBS). After 48 h of incubation, an Olympus microscope was used to measure the migration distance and calculate the migration rate. All experiments were repeated three times.

### Cell migration assay

The Transwell chamber (Corning) was used to evaluate migration in Transwell analysis. The cells were seeded in the upper cavity at a density of 5 × 10^4^/cavity, and RPMI-1640 containing 10% fetal calf serum was added as a chemotactic agent in the lower cavity. After 24 h of incubation at 37 °C, the cells in the upper chamber were removed with cotton swabs, and the cells in the lower chamber were fixed with formaldehyde for 30 min and stained with 0.1% crystal violet for 20 min. All experiments were repeated three times. Ten fields of view were randomly selected, and the positively stained cells were counted using an Olympus microscope.

### Cell invasion assay

The Transwell chamber was precoated (6.5 mm, Costar, Corning, NY, USA) with Matrigel for 30 min. Then, 1 × 10^5^ cells suspended in serum-free RPMI 1640 medium were inoculated into the upper chamber, and RPMI 1640 medium containing 10% FBS was added to the lower chamber. After incubating for 24 h at 37 °C, the cells in the upper chamber were removed with a cotton swab, the cells in the lower chamber were fixed with formaldehyde for 30 min, and stained with 0.1% crystal violet for 20 min. All experiments were repeated three times. Ten fields of view were randomly selected, and the positively stained cells were counted using an Olympus microscope.

### Three-dimensional culturing

Three-dimensional culture was used to evaluate the formation of VM. Fifty microliters of Matrigel (BD Biosciences, Sparks, MD, USA) was added to a 96-well plate and incubated at 37 °C for 30 min. Then, the cell suspension was coated on Matrigel and incubated at 37 °C for 8 h. A brightfield microscope was used to image five random fields of view of each well.

### Pseudopodia formation detection

The GC cells were seeded on glass slides for 24 h and cultured. The cells were then fixed with 4% paraformaldehyde and infiltrated with 0.3% Triton X-100. F-actin is an important pseudopodia component and is labeled with phalloidin labeled with Alexa Fluor 555 (A34055, Invitrogen, USA). Then the instructions were followed for Cortactin and DAPI (4ʹ,6-dimidyl-2-phenylindole) labeling. The slides were fixed with ProLongTM Gold Antifade Mountant (P36930 from Life Technologies, USA). FV3000 confocal laser scanning microscope (Olympus Japan) was used to obtain confocal micrographs under the control of supporting software.

### Xenograft and peritoneal dissemination model

Four-week-old female BALB/c nude mice were purchased from the Model Animal Research Center of Nanjing University and placed in a pathogen-free environment in the animal laboratory of Nanjing University. For the xenograft model, TNC knockdown and control GC cells (2 × 10^6^) were resuspended in 200 μL PBS. Then injected into the ventral side of nude mice (5 mice/group). Twenty-one days after tumor cell implantation, mice were sacrificed. The tumor was removed and weighed at autopsy. For the peritoneal diffusion model, 400 μL of TNC knockdown and control GC cells (3 × 10^6^) in PBS were injected into the peritoneal cavity. The peritoneal metastasis was checked and recorded when the mice were sacrificed on the 14th day after injection. No randomization was used and no blinding was done in this experiment.

### Statistical analysis

We used SPSS 22.0 (IBM) to statistically analyze the data. Three or more independent experiments are expressed as mean ± standard deviation. The comparison between the two groups was performed using the Student’s *t* test. One-way analysis of variance was used to compare the three groups. Counting data were compared by chi-square test. Linear regression was used to analyze the correlation between the two variables. The difference was considered statistically significant at a *P* value of <0.05.

## Results

### TNC was upregulated in gastric cancer tissues and correlated with poor prognosis in gastric cancer patients

**Fig. 1:**
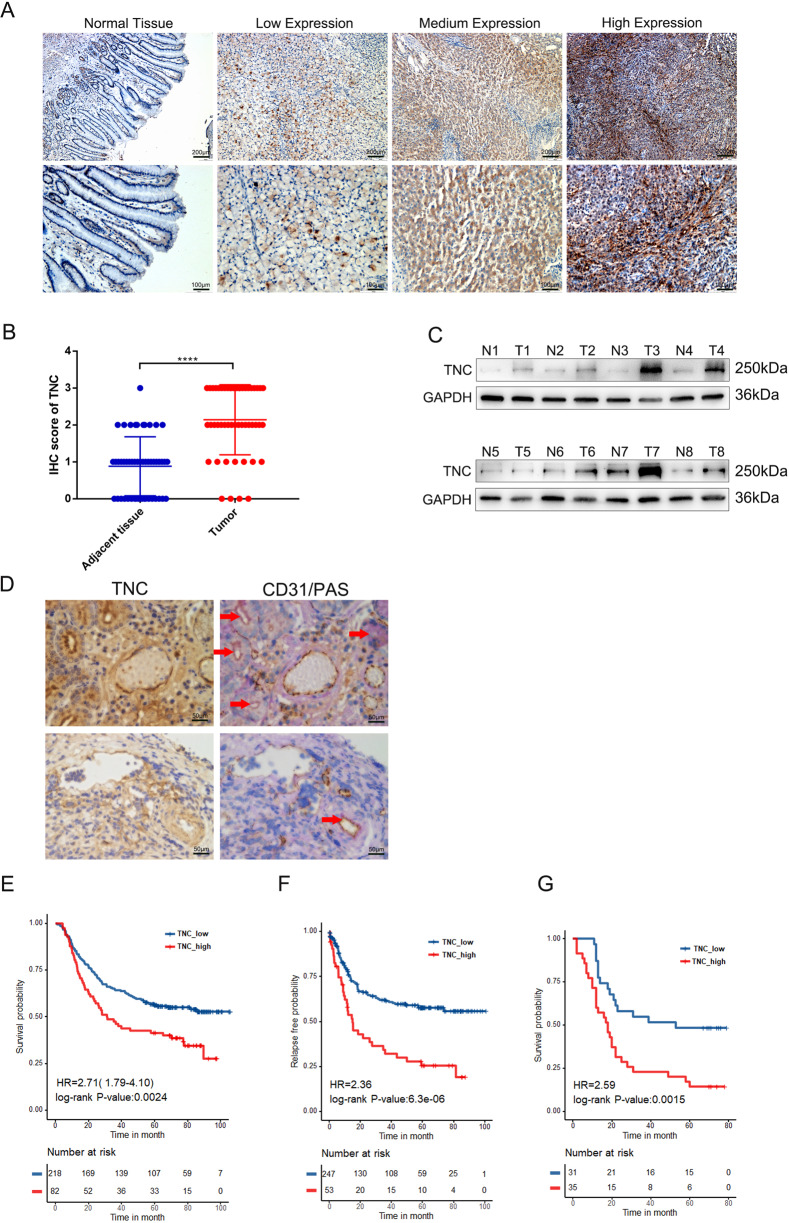
TNC was upregulated in gastric cancer tissues and correlated with poor prognosis in gastric cancer patients. **A** A representative IHC staining of normal and tumor tissue (magnification, ×100 and ×200). **B** TNC expression was significantly increased in gastric cancer tissues compared with adjust tissue. **C** Western blot analysis of TNC expression in gastric tissues and adjust tissue in eight patients. **D** IHC analysis of TNC, PAS and CD31 in gastric tissues; overall survival (**E**) and relapse-free survival (**F**) in patients with different TNC expression. Overall survival (**G**) of our single-center patients with different TNC expression. Results were shown as mean ± SD of three independent experiments; each experiment was performed in triplicate. ****P < 0.0001.

To evaluate TNC expression in gastric cancer tissues, we used immunohistochemistry to examine TNC expression in 50 pairs of gastric cancer tissues and corresponding adjacent tissues. TNC was mainly distributed in cytoplasm and extracellular matrix (Fig. 1A) and was significantly upregulated in cancer tissues than in normal tissues (Fig. 1B). Furthermore, we used western blot to examine the protein level of TNC in eight randomly selected gastric cancer tissues paired with adjacent tissues and demonstrated that TNC was significantly upregulated in gastric cancer tissues (Fig. 1C). Then, we used IHC to evaluate the TNC, PAS and CD31 expression in gastric cancer tissues. The results showed that VM exists in gastric cancer, consistent with previous studies [24, 25], and TNC expression had a positive correlation with VM in gastric cancer tissues (Fig. 1D). In addition, analysis of the association between TNC and prognosis of gastric cancer based on ACRG cohort revealed that patients with higher TNC levels had significantly shorter overall survival (OS) and relapse-free survival (RFS) compared to those with low TNC expression (Fig. 1E, F). Then we evaluated the expression of TNC in 66 postoperative specimens of gastric cancer patients and found that TNC expression was significantly correlated with tumor size, AJCC stage, T stage and N stage (Table 2). What’s more, Kaplan−Meier analysis also indicated that patients with high TNC expression had a shorter survival (Fig. 1G). These results indicated that the expression of TNC is correlated with cancer grade and may predict poor prognosis of gastric cancer patients.

**Table 2:** Clinicopathological information of patients with gastric cancer.

Variable	TNC expression		*P* value
	Strong (*n* = 35)	Weak (*n* = 31)	
Age at diagnosis (years)	63.19 ± 8.861	61.19 ± 8.199	0.242
Gender			0.092
Male	12	5	
Female	23	26	
Lauren classification			0.650
Intestinal	13	15	
Diffuse	10	7	
Mixed	12	9	
Tumor size (cm)			0.008*
<5	10	19	
≥5	25	12	
Tumor differentiation			0.308
Low	24	18	
Medium	10	10	
High	1	3	
AJCC stage			0.003*
I	1	8	
II	7	9	
III	27	14	
T stage			0.003*
T1	1	4	
T2	0	4	
T3	23	20	
T4	11	3	
N stage			0.011*
N0	6	13	
N1	5	7	
N2	5	2	
N3	19	9	
Nervous invasion			0.250
Positive	29	22	
Negative	6	9	
Venous invasion			0.082
Positive	30	21	
Negative	5	10	

*AJCC* American Joint Committee on Cancer, 8th edition.

*P < 0.05.

### Construction of cell lines and TNC knockdown attenuated VM formation in vitro

**Fig. 2:**
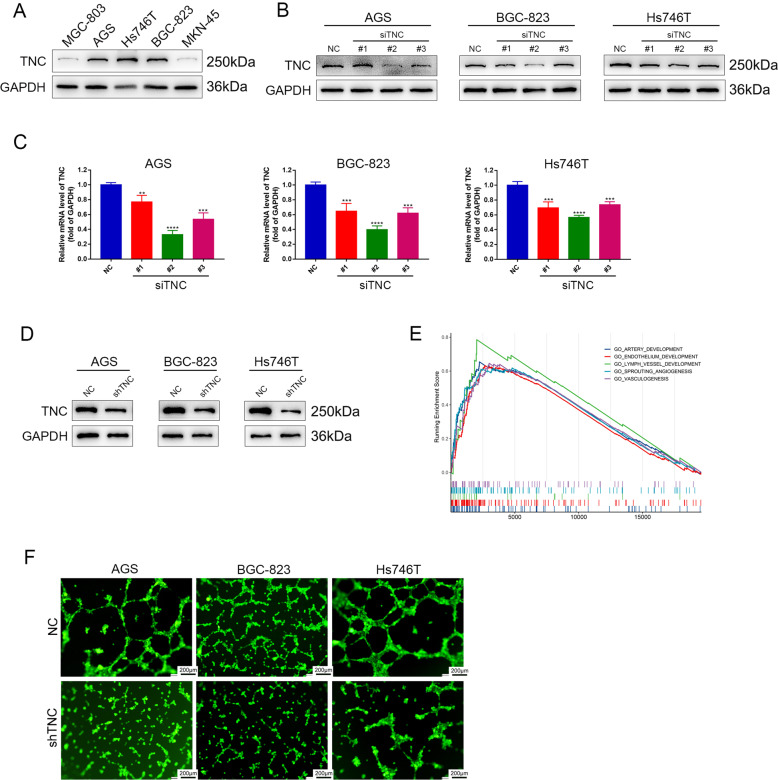
Construction of cell lines and TNC knockdown attenuated VM formation in vitro. **A** Western blot of TNC protein and gene expression in four gastric cancer cell lines; GAPDH was used as a loading control; protein (**B**) and gene (**C**) expression levels of TNC in AGS, BGC-823 and Hs746T cells transfected with siRNA targeting TNC. **D** Western blot analysis of knockdown efficiency of shTNC. **E** Bioinformation analysis of TNC functions. **F** VM was significantly reduced in AGS, BGC-823 and Hs746T cells after transfection with TNC shRNA. Results were shown as mean ± SD of three independent experiments; each experiment was performed in triplicate. **P < 0.01; ***P < 0.001; ****P < 0.0001.

We examined the expression of TNC by western blot in five gastric cancer cell lines, including MKN-45, AGS, Hs746T, BGC-823 and MGC-803 and found that TNC was highly expressed in AGS, BGC-823 and Hs746T cell lines (Fig. 2A). Thus, AGS, BGC-823 and Hs746T cells were selected to evaluate the efficiency of siRNA-mediated TNC knockdown. The transfection efficiency was confirmed by western blot and quantitative real-time PCR (Fig. 2B, C). The 2# siRNA was found to deliver the most effective knockdown and was used to construct lentivirus. The knockdown efficiency of lentivirus was examined by western blot (Fig. 2D). Then, we used bioinformatic analysis to explore the potential function of TNC and found that the function of TNC was strongly related to angiogenesis (Fig. 2E). Thus we boldly hypothesize that TNC is closely related to vasculogenic mimicry. Following this, we found that VM was significantly reduced in these cells after transfection with TNC shRNA (Fig. 2F).

### Knockdown of TNC inhibited the proliferation, migration and invasion of gastric cancer cells

**Fig. 3:**
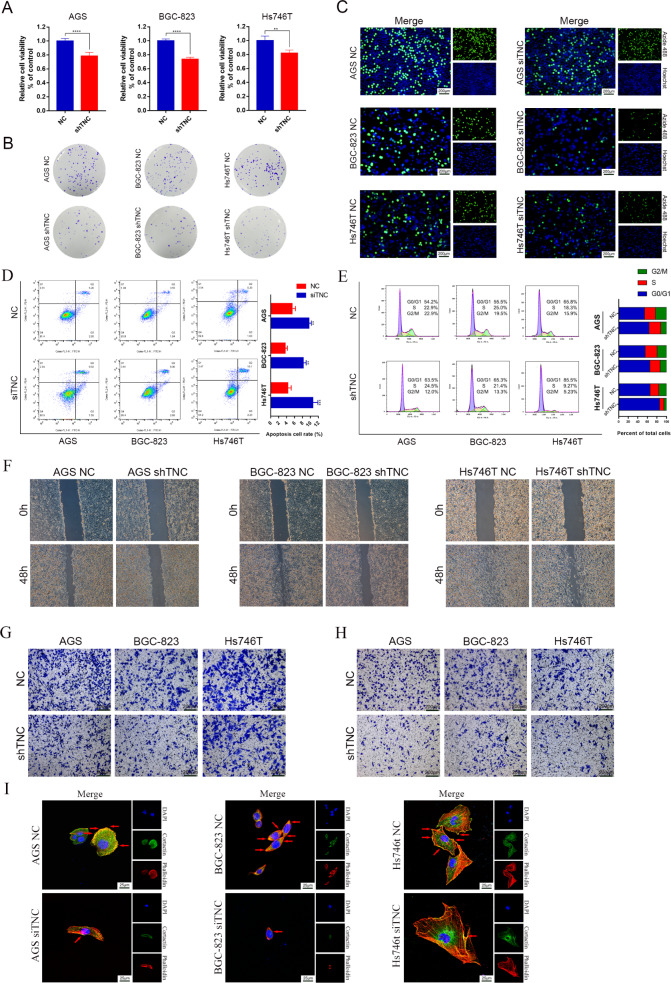
TNC knockdown inhibited the proliferation, migration and invasion of gastric cancer cells. CCK-8 (**A**), colony formation assay (**B**) and EdU assay (**C**) showed that TNC knockdown inhibited the proliferation of gastric cancer cells; flow cytometry showed that TNC induced cell cycle arrest (**D**) and apoptosis (**E**) in gastric cancer cells; cell migration was detected by wound-healing assay (**F**) and transwell assay (**G**); cell invasion was detected by transwell assay (**H**) and pseudopodia formation assay (**I**). Results were shown as mean ± SD of three independent experiments; each experiment was performed in triplicate. **P < 0.01; ****P < 0.0001.

Since VM formation is closely related to the proliferation, invasion and migration of tumor cells [26], we evaluated the detailed role of TNC in gastric cancer cells. CCK-8 assays showed that the viability of AGS, BGC-823 and Hs746T cells was significantly decreased in the shTNC group compared to the control group (Fig. 3A). Consistent with this, the colony formation assay (Fig. 3B) and EdU thymidine analog incorporation assay (Fig. 3C) also indicated that knockdown of TNC inhibited the proliferation of AGS, BGC-823 and Hs746T cells compared with the control group. What’s more, knockdown of TNC caused cell cycle arrest in the G2/M phase, preventing cell cycle progression from the S phase into the M phase in AGS, BGC-823 and Hs746T cells (Fig. 3D). Flow cytometry also showed that TNC knockdown increased Annexin V-positive cells in AGS, BGC-823 and Hs746T cells indicating that TNC knockdown induced apoptosis in gastric cancer cells (Fig. 3E). The migration of AGS, BGC-823 and Hs746T cells was evaluated through wound-healing (Fig. 3F) and transwell migration assays (Fig. 3G), indicating that TNC knockdown inhibited the migration rates of gastric cancer cells. Transwell assays (Fig. 3H) and pseudopodia formation assay (Fig. 3I) also revealed a significant reduction in invasion upon TNC knockdown.

### Knockdown of TNC inhibited ERK phosphorylation and EMT process

**Fig. 4:**
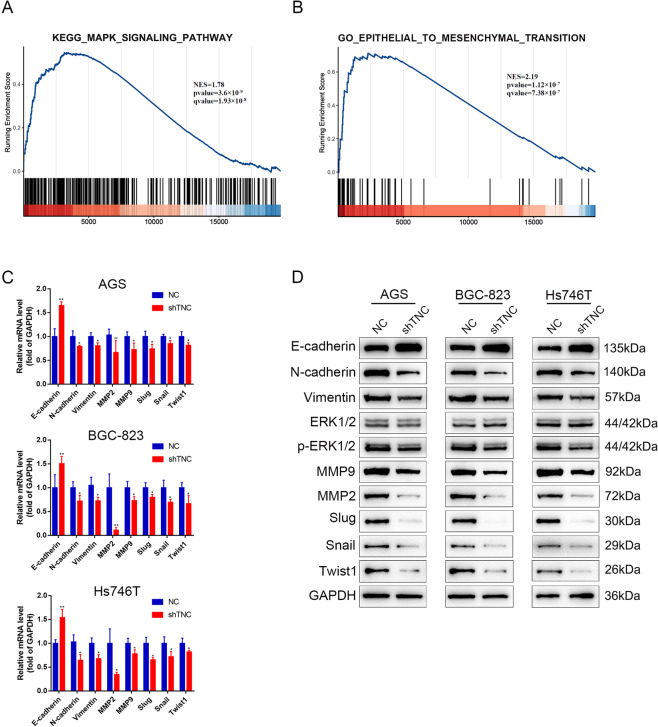
TNC correlated with ERK pathway and EMT process. **A** Gene Set Enrichment Analysis evaluating TNC expression and the MAPK_SIGNALING pathway in gastric cancer. **B** Bioinformatic analysis showed that TNC expression was in accordance with EMT process; qPCR (**C**) and western blot analysis (**D**) of ERK and EMT markers expression in AGS, BGC-823 and Hs746T cells transfected with TNC siRNA.

VM formation involves numerous factors including ERK, MMP2/9 and EMT process [9, 26–28]. Thus, we used Gene Set Enrichment Analysis to analyze the correlation between TNC expression in gastric cancer and activation of the MAPK pathway and found a significant positive association between TNC expression and activation of the ERK pathway (Fig. 4A). Bioinformatic analysis also showed that TNC expression was in accordance with EMT process (Fig. 4B). Subsequently, we examined the mRNA (Fig. 4C) and protein (Fig. 4D) levels of phospho-ERK, MMP2/9 and markers of EMT and found that phpspho-ERK, MMP2/9 were significantly decreased and gastric cancer cells was induced to proceed mesenchymal-to-epithelial transition process upon TNC knockdown.

### Knockdown of TNC inhibited VM through ERK-mediated EMT

**Fig. 5:**
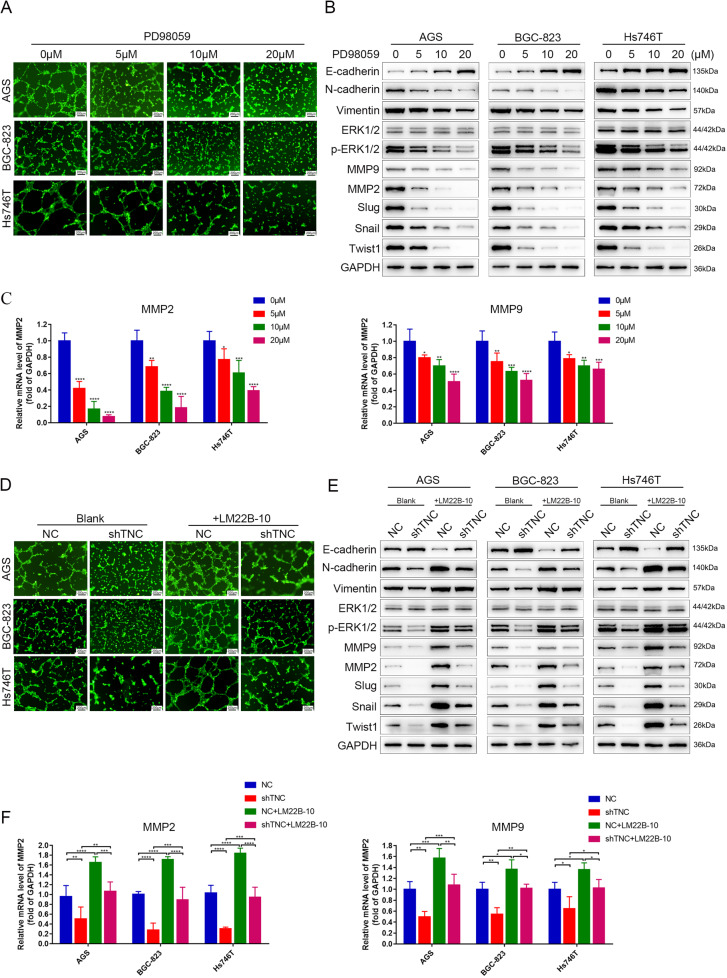
Knockdown of TNC inhibited VM through ERK-mediated EMT. **A** VM formation was inhibited in AGS, BGC-823 and Hs746T cells after exposure to the ERK inhibitor PD98059. **B** Western blot of ERK and EMT markers expression in AGS, BGC-823 and Hs746T cells after exposure to PD98059. **C** qPCR analysis of MMP2/9 in AGS, BGC-823 and Hs746T cells after exposure to PD98059. **D** VM formation of AGS, BGC-823 and Hs746T cells transfected with shTNC were increased upon treatment with the ERK activator LM22B-10. **E** Western blot of ERK and EMT markers expression in AGS, Hs746T and BGC-823 cells transfected with shTNC and treated with LM22B-10. **F** qPCR analysis of MMP2/9 in AGS, BGC-823 and Hs746T cells transfected with siTNC and treated with LM22B-10. Results were shown as mean ± SD of three independent experiments; each experiment was performed in triplicate. *P < 0.05; **P < 0.01; ***P < 0.001; ****P < 0.0001.

To determine whether ERK is associated with MMP2/9, EMT process and VM formation, PD98059, an ERK1/2 inhibitor, was used to conduct rescue experiment. As expected, VM formation of AGS, BGC-823 and Hs746T cells was reduced in a dose-dependent manner after treatment with 0, 5, 10 and 20 μM PD98059 for 24 h (Fig. 5A). What’s more, phosphor-ERK, MMP2/9, N-cadherin, Vimentin, Snail, Slug and Twist1 were downregulated and E-cadherin was upregulated in gastric cancer cells upon exposure to PD98059 at 0, 5, 10, 20 μM for 24 h (Fig. 5B, C). A rescue assay revealed that VM formation of AGS, BGC-823 and Hs746T cells transfected with shTNC was increased upon treatment with LM22B-10, an activator of ERK (Fig. 5D). The same changes were also observed in protein levels of phosphor-ERK, MMP2/9 and EMT markers (Fig. 5E, F), further supporting this conclusion.

### Knockdown of TNC inhibits tumor growth and peritoneal dissemination in vivo

**Fig. 6:**
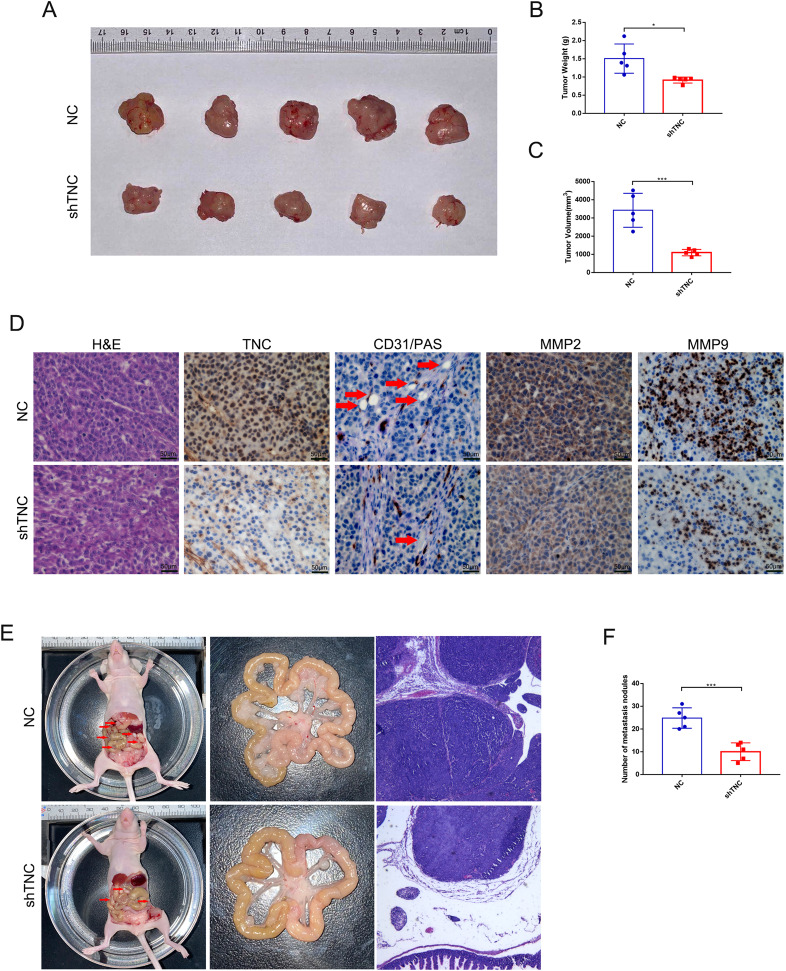
TNC knockdown inhibited tumor growth and peritoneal dissemination in vivo. **A** Image of tumors of mice in the NC and shTNC groups. **B** Volume of tumor of mice in the NC and shTNC groups. **C** Weight of tumor of mice in the NC and shTNC groups. **D** H&E, TNC, VM (CD31−/PAS+), MMP2 and MMP9 staining in NC and shTNC xenograft tissues. **E** Representative intestines in nude mice. **F** The quantification of metastases in nude mice was counted. Results were shown as mean ± SD of three independent experiments; each experiment was performed in triplicate. *P < 0.05; ***P < 0.001.

To determine the effects of TNC in vivo, xenograft and peritoneal dissemination model were established with AGS cells infected with lentivirus expressing shRNA-NC or shRNA-TNC. For xenograft model, 21 days after subcutaneous injection, mice were sacrificed, and the weight and volume of tumors were measured. The weight and volume of tumors in TNC knockdown group were decreased compared to the control group (Fig. 6A−C). Furthermore, TNC, VM (CD31−/PAS+), MMP2 and MMP9 in AGS xenograft tissues were significantly decreased in shTNC groups compared with that in the NC group (Fig. 6D). For peritoneal dissemination model, 14 days after peritoneal cavities injection, mice were sacrificed. The results showed that knockdown of TNC reduced the mesenteric metastatic nodules in the intestinal wall of nude mice (Fig. 6E, F).

## Discussion

Gastric cancer is one of the most common malignant tumors in the world [2] and recurrence and metastasis are the causes of the death in patients with gastric cancer. Once patients develop resistance to chemotherapeutics, anti-angiogenesis therapy becomes an important method to treat gastric cancer [29]. However, the effect of anti-angiogenesis therapy is not satisfactory and VM and metastasis continue to increase thereafter [30]. It has been reported that VM plays an important role in regulating the progression and metastasis in gastric cancer [11, 16]. However, the mechanism underlying VM formation in gastric cancer is unclear and it is urgent to seek therapies targeting VM formation.

TNC is an extracellular matrix protein and was reported to induce VM in glioma [26] and melanoma [31]. Recent studies demonstrated that TNC was strongly expressed and indicated poor prognosis in gastric cancer [16, 20]. The present results confirmed that TNC was upregulated in gastric cancer tissues and had a positive correlation with VN, and patients with higher TNC levels had significantly shorter OS and RFS compared to those with low TNC expression, consistent with our single-center data. Then, we evaluated VM formation in gastric cancer cell lines and found that TNC knockdown inhibited the VM formation ability in vitro and in vivo. Furthermore, knockdown of TNC suppressed the proliferation of gastric cancer cell lines and subcutaneous tumor and decreased the number of cells in the G0/G1 phase, indicating that TNC knockdown inhibited the proliferation of gastric cancer cell lines by inducing the cell cycle arrest in the G0/G1 phase. Concurrently, knockdown of TNC also inhibited the migration and invasion of gastric cancer cell in vitro and inhibited peritoneal metastasis in vivo.

To further investigate the mechanism underlying TNC and VM formation, we used bioinformatic analysis and found that TNC was positively associated with MAPK pathway and EMT process. As is known, EMT plays an important role in VM formation [9, 32] and MMP2/9 are important downstream effectors of ERK and contribute to VM formation [33]. Then, we investigated the correlation of TNC and VM with EMT markers in gastric cancer cells and found that TNC was positively correlated with N-cadherin, Vimentin, MMP2/9 and negatively related to E-cadherin, consistent with previous research [34, 35]. These results preliminarily indicated that TNC may promote VM formation by inducing ERK-triggered EMT. Then, we used PD98059 to inhibit ERK phosphorylation and found that VM and EMT process were decreased in a dose-dependent manner. What’s more, VM formation and EMT process of TNC knockdown cells were reversed by LM22B-10, further proving the hypothesis.

In conclusion, our results demonstrated that TNC knockdown blocked the EMT process by suppressing the ERK pathway, leading to the inhibition of VM formation. The combined targeting of TNC and ERK pathway may provide a potential antitumor therapy for inhibiting VM formation and decrease antiangiogenic therapeutic resistance.

## Data Availability

The data supporting our conclusion were obtained from the TCGA database (https://cancergenome.nih.gov). All the data supporting the conclusions were included in the main paper.

## Materials availability

All the materials supporting the conclusions were included in the main paper.
