# Correction to: Tenascin-c knockdown suppresses vasculogenic mimicry of gastric cancer by inhibiting ERK- triggered EMT

**DOI:** 10.1038/s41419-021-04456-3

**Published:** 2021-12-13

**Authors:** Xing Kang, En Xu, Xingzhou Wang, Lulu Qian, Zhi Yang, Heng Yu, Chao Wang, Chuanfu Ren, Yizhou Wang, Xiaofeng Lu, Xuefeng Xia, Wenxian Guan, Tong Qiao

**Affiliations:** 1 Department of General Surgery, Nanjing Drum Tower Hospital Clinical College of Nanjing Medical University, No. 321 Zhongshan Road, 210008 Nanjing, China; 2 Department of General Surgery, Affiliated Drum Tower Hospital, Medical School of Nanjing University, No. 321 Zhongshan Road, 210008 Nanjing, China; 3 Department of Vascular Surgery, The Affiliated Drum Tower Hospital of Nanjing University Medical School, No. 321 Zhongshan Road, 210008 Nanjing, China

**Keywords:** Gastric cancer, Tumour angiogenesis

Correction to: *Cell Death & Disease* 10.1038/s41419-021-04153-1, published online 29 September 2021

The original version of this article unfortunately contained a mistake in the author affiliations. Due to the authors carelessness, the corresponding author “Tong Qiao” missed a superscript. The authors apologize for the mistake. The original article has been corrected.

